# Comparison of the rate of delayed/nonunion in fifth metatarsal fractures receiving anti-inflammatory medications

**DOI:** 10.1186/s40634-021-00435-x

**Published:** 2021-12-11

**Authors:** Brandon Barnds, Matt Heenan, Jack Ayres, Armin Tarakemeh, J. Paul Schroeppel, Scott Mullen, Bryan G. Vopat

**Affiliations:** Department of Orthopaedics, University of Kansas Medical Center, Kansas City, KS 66160 USA

**Keywords:** Jones Fracture, Nonunion, NSAIDs, 5^th^ Metatarsal, Anti-inflammatory

## Abstract

**Purpose:**

Controversy exists regarding the acute effect of non-steroidal anti-inflammatory drugs (NSAIDs) on early fracture healing. The purpose of this study was to analyze the rate of nonunion or delayed union in patients with fifth metatarsal (5^th^ MT) fractures. We hypothesize that the use of NSAIDs would increase the rate of nonunion/delayed union in 5^th^ MT fractures.

**Methods:**

Using PearlDiver, a national insurance database was analyzed. ICD codes were used to identify patients diagnosed with 5^th^ MT fracture from 2007-2018. Patients were grouped by initial management (nonoperative vs. open reduction and internal fixation (ORIF) or non/malunion repair within 60 days) and sub-grouped by whether they had been prescribed at least one pre-defined NSAID. Subsequent ORIF or nonunion/malunion repair operative intervention was used as a surrogate for fracture nonunion/delayed union.

**Results:**

Of the 10,991 subjects with a diagnosis of 5^th^ MT, 10,626 (96.7%) underwent initial nonoperative treatment, 1,409 of which (13.3%) received prescription NSAIDS within 60 days of diagnosis. 16/1,409 (1.14%) subjects who received anti-inflammatory prescriptions underwent ORIF or repair of non/malunion at least 60 days after diagnosis while 46/9,217 (0.50%; *P=0.003483*) subjects who did not receive anti-inflammatory prescriptions underwent ORIF or repair of non/malunion at least 60 days after diagnosis. In the 365 subjects who underwent early repair/ORIF (within 60 days), there was no significant difference in the rate of nonunion/delayed union.

**Conclusion:**

The rate of nonunion/delayed union of 5^th^ MT fractures was significantly higher in subjects receiving NSAIDs within 60 days of initial diagnosis in patients managed non-operatively.

**Level of evidence:**

Level III

## Introduction

The occurrence of nonunion and delayed union of fractures has significant implications in the care of orthopedic trauma patients. This is a multifactorial phenomenon requiring investigation to limit modifiable causes including administration of medications which may increase the rate of nonunion [15]. Non-steroidal anti-inflammatory drugs (NSAIDs) are often used to treat pain, with over 25% of the population endorsing that they utilize NSAIDs [4]. Controversy exists regarding the acute effect of NSAIDs on early fracture healing. Recent reviews have highlighted this variability and have concluded that no consensus on the effect of NSAIDs on fracture healing can be made [14].

NSAIDs are known to reduce inflammation by inhibiting cyclooxygenase (COX)-1 and -2, resulting in downregulation of prostaglandins, leading to the theory that these common medications can alter the inflammatory phase of fracture healing [13]. Specifically, COX-2 is necessary for differentiation of mesenchymal cells to osteoblasts and thus these medications can reversibly suppress osteoblast formation [14, 16, 17]. Additionally, in-vitro studies have demonstrated the ability of common NSAIDs including Indomethacin to reduce osteoclast activity and mineral deposition in animal cell lines [11]. Animal models have largely demonstrated that fracture union is impaired by NSAIDs when assessing callus formation, stiffness, and thickness, leading to an increased rate of delayed union and nonunion [1, 7].

Fifth metatarsal (MT) fractures in general are the most common MT fractures experienced by adults and adolescents, affecting both the young athletic population and middle-aged patients, with an average age at injury of 51 [10, 20]. The majority of these fractures involve the proximal 1/3 of the 5^th^ MT (70-71%) and 17% involve the shaft [10, 12]. The location and patient characteristics greatly change the treatment recommendation: shaft and distal fractures are often treated nonoperatively while there is significant variability in the treatment of proximal fractures. To help determine optimal treatment, proximal fractures are classified as zone I (tuberosity), zone II (metaphyseal-diaphyseal junction, within 1.5 cm of the tuberosity), and zone III (proximal diaphysis distal to the 4-5th MT articulation) [5, 12, 20]. In-part due to the vascular watershed characteristics of zone II fractures, the nonunion rate in zones II fractures managed nonoperatively ranges from 20-30%, with a mean time to radiographic union of 15.9 weeks [5, 12, 18]. Therefore, some authors suggest surgical fixation with percutaneous intramedullary screw placement for zones II and III fractures [5, 12, 20]. This is especially recommended for athletes desiring a quicker return to play, as the average time to union is 8-10 weeks and is associated with lower rates of both nonunion and refracture [5, 12, 20]. Overall, however, there is a lack of consensus on operative versus non-operative management as has been reported in several studies and reviews, [5, 21, 22] and the treatment decision may consist of fracture classification as well as numerous other factors such as patient demographics [2].

Due to the relatively high rate of nonunion in 5^th^ metatarsal fractures (specifically Zone II), it is important to mitigate factors that decrease healing in this fracture. NSAIDs are one type of common medication that has been associated with impeding bone formation in other parts of the body. We hypothesize that the use of NSAIDs would increase the rate of nonunion/delayed union requiring surgical fixation in 5^th^ metatarsal fractures using a private payer database.

## Methods

This study was conducted using the PearlDiver Health Insurance Database (PearlDiver, Colorado Springs, CO). Specifically, this study utilized the Humana Insurance Database of over 25 million orthopedic patients from 2007-2018. Patients with a history of 5^th^ metatarsal fracture (ICD-10-D-S92351 through ICD-10-D-S92355) were isolated using a customized PearlDiver code. Patients having undergone ORIF or repair of non/malunion of MT acutely within 60 days of diagnosis were included in the group initially managed operatively based on having a concomitant Current Procedural Terminology (CPT) code for these procedures. A second treatment group included patients having a 5^th^ MT fracture managed nonoperatively after initial diagnosis. Two sub-groups were then created based on whether patients received an outpatient prescription for at least one of a group of common NSAIDs (Table 1) [9, 14].

**Table 1:** NSAIDs Analyzed

Ibuprofen	
Flurbiprofen	
Ketorolac	
Piroxicam	
Indomethacin	
Meloxicam	
Celecoxib	
Diclofenac	
Naproxen	
Nabumetone	

Patients in both groups were then followed for progression to delayed ORIF or non/malunion repair based on having a CPT code for these procedures (CPT-28322 and CPT-28485) greater than 60 days after initial diagnosis. This analysis created a subgroup of patients experiencing delayed union or nonunion of 5^th^ MT fractures that went on to operative treatment or reoperation for nonunion and a subgroup who did not require an operation or reoperation for nonunion, representing patients who did not experience a clinically symptomatic nonunion/delayed union. The timing of 60 days was determined based off findings in the literature of nondisplaced 5^th^ MT fractures treated non-operatively healing as soon as 6 to 8 weeks (42 to 56 days) after injury [18].

The rate of delayed ORIF and malunion/nonunion repair was used as a surrogate for nonunion or delayed union and was compared between the NSAID and non-NSAID groups, given that surgical treatment is the standard of care for 5^th^ MT nonunion or delayed union [19]. A chi-square analysis was performed to assess significance. A p value < 0.05 was considered significant.

## Results

Initial query resulted in 10,991 subjects with a diagnosis of 5^th^ MT fracture identified in the database. 365 subjects underwent early ORIF (within 60 days) and were included in the group initially managed with ORIF, leaving 10,626 initially managed nonoperatively (Table 2).

**Table 2:** Demographics of Subjects Initially Managed Nonoperatively

	Non-NSAIDS		NSAIDS	
	No Surgery	Surgery	No Surgery	Surgery
Total	9171	46	1393	16
Sex n (%)				
Male	2167 (23.6)	7 (15.2)	288 (20.7)	<11
Female	7004 (76.4)	39 (84.8)	1105 (79.3)	<11
Age				
<10	68 (0.7)	0	<11	0
10-14	438 (4.8)	0	17 (1.2)	0
15-19	189 (2.1)	<11	<11	0
20-29	262 (2.9)	<11	44 (3.2)	0
30-39	337 (3.7)	<22	48 (3.4)	<22
40-49	541 (5.9)	<11	141 (10.1)	<22
50-59	1138 (12.4)	<22	293 (21.0)	<22
60-69	2335 (25.5)	<22	415 (29.8)	<22
70-79	2525 (27.5)	<22	315 (22.6)	0
80-89	1114 (12.1)	<11	93 (6.7)	0
>90	224 (2.4)	0	14 (1.0)	0
Region				
Midwest	1994 (21.7)	<11	221 (15.9)	<11
Northeast	222 (2.4)	<11	24 (1.7)	0
South	6083 (66.3)	29 (63.0)	1053 (75.6)	<11
West	872 (9.5)	<11	95 (6.8)	<11
CCI average (standard dev.)	1.88 (2.69)	2.13 (2.75)	1.76 (2.49)	0.56 (0.89)

In the group initially managed nonoperatively, 1,409/10,626 (13.3%) received an outpatient prescription for NSAIDs within 60 days of diagnosis, leaving 9,217/10,626 (86.7%) who did not receive these prescriptions (Table 3). 16/1,409 (1.14%) subjects who received NSAID prescriptions underwent ORIF or repair of non/malunion at least 60 days after diagnosis. 46/9,217 (0.50%; *P=0.003483*) subjects who did not receive these prescriptions underwent ORIF or repair of non/malunion at least 60 days after diagnosis.

**Table 3:** Patients with 5^th^ MT Fractures Initially Managed Nonoperatively

	Total	NSAIDs	No NSAIDs	P-value
Number of Patients	10,626	1,409 (13.3%)	9,217(86.7%)	
Delayed ORIF or Malunion/Nonunion Repair		16 **(1.14%)**	46 **(0.50%)**	**0.003483**

Analysis of the group initially managed operatively with ORIF in the first 60 days after diagnosis yielded the following: 68/365 (18.6%) received an outpatient prescription for NSAIDs within 60 days of diagnosis, leaving 297/365 (81.4%) who did not receive these prescriptions (Table 4). 1/68 (1.5%) of subjects who received NSAID prescriptions underwent secondary ORIF or repair of non/malunion at least 60 days after initial ORIF, whereas 5/297 (1.7%, *P=0.900876*) of patients who did not receive an NSAID prescription underwent a secondary ORIF or repair of non/malunion repair.

**Table 4:** Patients with 5^th^ MT Fractures Initially Managed Operatively

	Total	NSAIDs	No NSAIDs	P-value
Number of Patients	365	68 (18.6%)	297 (81.4%)	
Delayed ORIF or Malunion/Nonunion Repair		1 (1.5%)	5 (1.7%)	0.900876

## Discussion

The most important finding from this study demonstrated that the use of NSAIDs increased the rate of delayed union/nonunion in patients treated initially nonoperatively for 5^th^ metatarsal fractures. In the group initially managed nonoperatively, the rate of delayed union/nonunion as evidenced by undergoing ORIF or non/malunion repair greater than sixty days after initial diagnosis was found to be 16/1,409 (1.14%) in the NSAID group and 46/9,217 (0.50%; *P=0.003483*) in the non-NSAID group. However, we did not find a difference in non/malunion rate in patients who were treated with initial operative intervention. In the group initially managed with early ORIF, 1/68 (1.5%) of subjects who received NSAID prescriptions required secondary surgeries compared to 5/297 (1.7%, *P=0.900876)* in the non-NSAID group.

There has been considerable research utilizing in-vitro cellular models and animal models to determine the effects of NSAIDs on the inflammatory cascade, fracture healing, and cellular modification. Given the difficulty in controlling over-the-counter use of NSAIDs, the wide range of medications falling under the NSAID classification, and the power necessary to assess a small but potentially important effect on nonunion rate, there are few level 1 randomized controlled trials assessing the true risk of nonunion or delayed union in patients receiving NSAIDs [14]. Thus, some studies have utilized databases to retrospectively assess the effect of NSAIDS on the rate of fracture complications including nonunion, delayed union, malunion, or infection, most of these studies having no more than a few hundred patients [14]. The current study is the first of its kind to use a large insurance database to specifically assess the effects of NSAIDs on 5^th^ MT fracture healing.

Retrospective studies have demonstrated an increased rate of nonunion or delayed union in patients experiencing a fracture who are treated with NSAIDs. A study by Giannoudis et al. [8] retrospectively reviewed 377 patients with a femoral shaft facture managed with intramedullary nailing, finding a higher rate of nonunion in patients treated with ibuprofen and/or diclofenac. Specifically, 62.5% of patients with a nonunion had received NSAIDs versus 13.4% of patients who did not have nonunion [8]. Additionally, the average time to union was 7.5 months in the NSAID treated group and 5.5 months in the non-NSAID group [8]. Furthermore, a large retrospective insurance database study analyzing a total of 309,330 patients with fractures demonstrated a higher rate of nonunion in patients prescribed NSAIDs with an odds-ratio of 1.84 [23]. Therefore, these large database studies are demonstrating the potentially detrimental effects of NSAIDs on fracture hea ling. The current study adds to the body of literature demonstrating the same for an isolated foot fracture initially managed nonoperatively with a significantly increased rate of clinically relevant nonunion/delayed union in patients managed with NSAIDs (1.14% in the NSAID group vs 0.50% in the non-NSAID group (*P*=0.003483)).

Furthermore, while this study includes pa tients of all ages (Table 2), and does not delineate outcomes based on age groups, there is literature suggesting that NSAIDs do not have the same detrimental effect on fracture union in pediatric models. Specifically, large retrospective studies of pediatric patients with fractures managed nonoperatively by the emergency department have demonstrated no effect of Ibuprofen on nonunion [6]. Furthermore, juvenile animal models of tibial fractures exposed to Ketorolac demonstrated no significant reduction in strength and stiffness on mechanical testing [3]. The purpose of this study was to use a private payer database to assess the effects of NSAIDs on 5^th^ MT fracture healing.

This study is limited in the retrospective nature of its design. This study relies upon accurate provider coding and billing information, and the use of a private, single payer database. The use of a private payer database may have biased our results; however, the authors were reassured by the large sample size with demographics as represented in Table 2. The coding systems used in this analysis do not specify the zone of fracture, which may be an important consideration. Additionally, due to the nature of the design, the study was unable to determine which patients received an NSAID while inpatient or those taking NSAIDs over-the-counter. It was also unable to determine which patients received a prescription but did not take the NSAID. Finally, it lacks any subjective outcome measures, and patients may have changed insurance companies resulting in loss to follow up.

## Conclusion

In conclusion, the rate of delayed ORIF or non/malunion repair of 5^th^ MT fractures, a surrogate for delayed union or nonunion in an insurance database study, was significantly higher in subjects receiving anti-inflammatories w ithin 60 days of initial diagnosis for patients initially managed non-operatively. The hypothesis that NSAIDs would have a negative effect on 5^th^ MT fracture union was supported in patients initially managed non-operatively. NSAIDs are a negative prognostic factor for 5^th^ metatarsal fracture healing when managed non-operatively.

## Abbreviations

COX: Cyclooxygenase
CPT: Current Procedural Terminology
ICD-9: International Classification of Diseases, Ninth Revision
ICD-10: International Classification of Diseases, Tenth Revision
MT: Metatarsal
NSAID: Non-steroidal anti-inflammatory drug
ORIF: Open reduction and internal fixation
